# 

**Published:** 1887-01-15

**Authors:** 


### THE HOSPITAL

An Institution, Family, and Parochial Journal of
Hospitals, Asylums, & all Agencies for the Care of the Sick, Criticism & News.

### CONTENTS.

**Child Torture in Christian England** - - 257
**Words of Consolation.**—XVI. Sympathy - - 258
**A Good Work Well Finished.**—"Finis Coronat Opus" - - 259
**The Story of the Hospitals.**—The British Home for Incurables - - 260
**Lancaster Town: its Mayor and its Hospital.** By Our Special Commissioner - 263
**Notes and News.**—The Duke of Westminster and the Hospital for Women, Soho.—Miscellaneous Items.—Presentation of Busts to the Manchester Royal Infirmary.—Vacancies.—Hospital Enlargements.—Retirement of Mrs. Rahn.—The Norwich Hospital Sunday Fund Collection.—Chard and the Jubilee.—The "Elephant Man."—Edinburgh Royal Infirmary Report.—Income of London Hospitals.—Towns without Hospitals.—Hospital Bazaar at Hastings.—The Edinburgh Provident Dispensary.—The Founder of Guy's Hospital.—Dramatic Entertainments in aid of the Hospitals.—The Nursery Hospital at Nottingham.—Christmas Entertainments at the Hospitals.—The Newcastle Infirmary.—Improvements at the Suffolk General Hospital, etc. - 264
**Till the Doctor Comes.**—VIII. Practical Hints for the Sick-room - - 269
**More Christmas Festivities at the Hospitals.** By Our Special Commissioner.—The Brompton Consumption Hospital.—The London Hospital.—The Evelina Hospital for Children - 269
**A Serious Question** - - 271
**The Children's Corner.**—Puzzles, Answers, etc. - 272
**The Hospital Charity Box** - - 272
**In- and Out-Patients' Hospital Diary** - - 273

